# A potential synbiotic product improves the lipid profile of diabetic rats

**DOI:** 10.1186/1476-511X-11-114

**Published:** 2012-09-11

**Authors:** Mariana N Roselino, Nadiége D Pauly-Silveira, Daniela CU Cavallini, Larissa S Celiberto, Roseli A Pinto, Regina C Vendramini, Elizeu A Rossi

**Affiliations:** 1 Department of Food & Nutrition, School of Pharmaceutical Sciences, Sao Paulo State University, Araraquara, SP, Brazil; 2 Department of Clinical Analysis, School of Pharmaceutical Sciences, Sao Paulo State University, Araraquara, SP, Brazil

**Keywords:** Fermented soy product, Probiotics, Prebiotics, Diabetes mellitus, Blood glucose, Lipid profile

## Abstract

**Background:**

Previous studies showed that intake of yacon or some lactic acid bacteria was able to inhibit the development of diabetes mellitus, by reducing glucose and associated symptoms, for example, the lipid profile.

**Objective:**

The purpose of this study was to assess the consumption influence of a potential symbiotic product of soybean and yacon extract and fermented *Enterococcus faecium* CRL 183 and *Lactobacillus helveticus* ssp *jugurti* 416 in reducing blood glucose and lipid levels in an animal model.

**Methods:**

Diabetes mellitus was chemically induced by intraperitoneal administration of streptozotocin (50 mg/kg body weight). The rats were divided into four groups (n=10): GI – non-diabetic animals that received only a standard chow diet (negative control), GII – diabetic animals that received only chow diet (positive control), GIII – diabetic animals that received the chow diet + 1 mL/kg body weight/day of soybean and yacon unfermented product, GIV – diabetic rats that received the chow diet + 1 mL/kg body weight/day of soybean and yacon fermented product. There was a seven-week treatment period and the following parameters were evaluated: animal body weight, food and water intake, blood glucose, enzyme activities of aspartate aminotransferase (AST) and alanine aminotransferase (ALT), triglycerides levels, total cholesterol, HDL-C, non-HDL-C. Cell viability of the fermented product was checked weekly for a seven-week period.

**Results:**

The product average viable population was 10^8^-10^9^ CFU/mL, by ensuring both the rods and cocci regular intake. No difference was observed between the water and feed intake and body weight of groups that received unfermented and fermented products and the untreated diabetic group. The same was observed for the blood glucose and AST and ALT activities, while some improvement was observed for a lipid profile, represented by reduction of triglycerides level by 15.07% and 33.50% in groups III and IV, respectively, and an increase of 23.70% in HDL-C level for group IV.

**Conclusion:**

The results showed that the ingestion of a potential symbiotic product was neither able to promote improvement in some of the disease symptoms, nor reduce blood glucose. However, a positive effect on triglycerides levels and HDL-cholesterol was observed in the groups that received the unfermented product containing yacon extract and the fermented product with *Enterococcus faecium* CRL 183, as well as *Lactobacillus helveticus* ssp *jugurti* 416 and yacon extract (symbiotic product).

## Background

Diabetes mellitus is among the tenth leading causes of death in western countries and, despite progress in its clinical management, it is not yet possible to control its lethal consequences. This disease is a chronic disorder that affects the carbohydrates metabolism, fats and proteins. The characteristic of diabetes mellitus is hyperglycemia, which reflects a deterioration in the use of glucose (and hence carbohydrates in general) due to defective insulin secretion or deficient response to it [1,2]. Diabetes mellitus comprises a number of common symptoms, such as excessive thirst and hunger, weakness, weight loss and rising blood glucose, resulting in excretion of glucose in the urine [3-7]. According to Mori et al. [8], diabetes mellitus is capable of reproducing the hepatic toxic characteristics, increasing the AST and ALT levels in serum blood, therefore, being useful as hepatic biomarkers.

The atherosclerosis risk is twice to three times greater in diabetics than in non-diabetics. The absence of insulin promotes hydrolysis of stored triglycerides in the adipocytes and their presence in the circulation, and the leading to a reduction in serum levels of HDL-C [9-11].

The probiotic foods are those that contain microorganisms that modulate the intestinal microbiota and aid the functioning of the gastrointestinal tract and thus may prevent the disease, while prebiotics are food containing substances which are resistant to enzymatic breakdown, which stimulate the proliferation or activity of certain bacteria in the intestinal microbiota, acting as a selective substrate in the colon. Foods that have both probiotics and prebiotics are called synbiotics [12].

Within this context, fermented foods containing probiotics and prebiotics can be important diet components, due to their nutritional characteristics and ability to reduce the risk of chronic diseases [13-17].

In recent decades, the yacon (*Smallanthus sonchifolius*), a natural plant in the Andean region, has aroused the interest of researchers as it provides bioactive compounds considered important for human health [18]. In Bolivia, it is the potato - like yacon roots are commonly consumed by people suffering from diabetes [19]. In studies of the fructooligosaccharides and inulin found in yacon, lipid-lowering effects have been observed [20].

It has also been reported that some lactic acid bacteria affect the progression of diabetes mellitus [21-25]. These studies show that ingestion of determined lactic acid bacteria prevents or delays the disease onset in various experimental models of diabetes, induced by a chemical or by diet, or genetically modified animals (dB/dB). However, studies relating the use of lactic acid bacteria to the development of diabetes are scarce [26]. A variety of *in vitro* experiments and *in vivo* trials have provided experimental evidence to support the probiotic roles in lowering serum cholesterol and improving lipid profiles [27].

Considering the probable hypoglycemic effect of yacon, the soybean health beneficial effects and the probiotic lactic acid bacteria [28-32], it seemed timely and of great interest to assess the consumption influence of a potential synbiotic product of soybean and yacon extract and fermented with *E. faecium* CRL 183 and *L. helveticus* ssp. *jugurti* 416 in reducing blood glucose and lipid levels in a streptozotocin-induced diabetes mellitus in rats.

## Materials and methods

### Animals and experimental protocol

Male wistar rats (n=40), aged 5 weeks (weight 130-170 g), were obtained from the Central Animal Facility of the Campus of Botucatu -UNESP, SP, Brazil. During the 59-day experiment, the animals were kept under controlled temperature (22°C ± 2°C), with a light–dark cycle of 12:12 h, with free access to standard chow and water. The product under study was administered to the animals for seven weeks.

Rats were fed a standard chow diet (Purina®-SP, Brazil) for a week, to acclimatize them, and then they were randomly allocated to four experimental groups (n=10): GI - non-diabetic animals that received only the chow diet (negative control); GII - diabetic animals that received only chow diet (positive control); GIII – diabetic animals that received chow diet + 1 mL/kg body weight/day of soybean and yacon extract unfermented product; GIV – diabetic rats that received chow diet + 1 mL/kg body weight/day of soybean and yacon extract fermented product.

The fermented product, based on aqueous extracts of soybean and yacon, was processed at the Development and Production Unit for Soybean Derivatives (UNISOJA), Food and Nutrition Department of the School of Pharmaceutical Sciences, UNESP in Araraquara, SP, Brazil, by methods described by Pauly-Silveira [33], but all the sugar was replaced by sucralose, because of the diabetic groups condition who consumed the product.

The Foscarin soybean variety (Ribeirão Preto, SP, Brazil) was used in the soy milk processing (raw material of unfermented and fermented products). The hulled grains were submitted to a heat treatment (95°C/7 min) and led to a continuous extracting machine (Perfecta®, Curitiba, PR, Brazil) to obtain the soy milk. The used yacon was of yellow variety and was purchased in local marketing. Immediately after yacon cutting, the pieces were blanched in hot water (97°C) and citric acid was added, for a 15-min period. Finally, the yacon was ground and filtered to obtain the aqueous extract [33]. The bacterial inocula consisted of 3.0% (v/v) of a 1:1 mixture of *Enterococcus faecium* CRL 183 (probiotic microorganism from CERELA - Argentina) and *Lactobacillus helveticus* ssp. *jugurti* 416 (adjuvant fermentation from Institute of Food Technology - ITAL - Campinas, SP, Brazil). The aqueous extracts mixed at soybean rates: yacon of 60.00% to 25.86% [33]. The fermentation was conducted at temperatures of 37°C until the pH of 4.5 was reached. The unfermented and fermented products showed the following proximal composition (%): moisture 82.85; total solids 17.15; protein 3.97; fat 2.49; ash 0.51; carbohydrates 10.19.

The unfermented product was prepared by chemical acidification of the synbiotic product base mixture (without bacterial culture) with food-grade lactic acid (Purac, Sao Paulo, Brazil), to reach the same pH as the fermented product (4.4 - 4.6).

After the period of adaptation, the rats were fasted for 12 h. Afterwards, the rat group II; then, the groups III and IV by injections (intraperitoneal) of 50 mg per kg body weight of streptozotocin (STZ) (Sigma Aldrich®, St Louis, MO, USA) [34,35] dissolved in 10 mM citrate buffer (pH 4·5), while animals of group I received a similar buffer injection. Three days later, the blood glucose level of STZ-treated rats had risen to 500 mg/dL, while group I remained around 120 mg/dL.

The unfermented and fermented administration products started after confirmation of diabetes induction. The products were administrated to the animals by gavage of 1 mL/kg body weight once every day, to ensure a minimal intake of 10^7^ CFU/day of *E.faecium* and of *L.helveticus* ssp *jugurti*, in the fermented products for a seven-week period. The fermented product contained 10^8^**-** 10^9^ CFU/mL of each microorganism.

The experimental design received approval from the Research Ethics Committee of the School of Pharmaceutical Sciences, UNESP in Araraquara, SP, Brazil (protocol CEP/FCF no. 21/2010)

### Measurement of total fructans

The total fructan content was measured in triplicate by a spectrophotometric method and was carried out in yacon extract, in the unfermented and potential synbiotic products (Amersham Biosciences® model Ultrospec 3100 pro, USA) using the Megazyme Fructan HK Assay kit (AOAC Method 999.03 and AACC Method 32.32; Megazyme International Ireland Ltd., Wicklow, Ireland) [36].

### Body weight, food and water intake

The body weight of each animal was recorded weekly throughout the experimental period.

The feed and water intakes of each animal were determined by weight difference between chow offered and orts and the difference between the amounts of water offered from remaining bottles, respectively, and recorded daily throughout the experimental period.

### Sample collection

Blood samples were collected from a small longitudinal incision made at the animal tail end [37].

### Glycemia

The level of glucose in the plasma was assessed at the beginning of the experiment, three days after induction of diabetes, to verify the diabetic state, 14 days after proof of the diabetic state and at the end of the experiment, after the final administration of the products, using Labtest Diagnostic® Kit SA (Lagoa Santa, MG, Brazil) [38,39].

### Liver enzymes (aspartate aminotransferase and alanine aminotransferase)

The enzymatic activity of the transaminases AST and ALT was determined in the plasma by Labtest Diagnostic® Kit SA (Lagoa Santa, MG, Brazil), at the same time as the glucose analysis [38,39].

### Serum lipids

Lipids were determined in the plasma, by using Labtest Diagnostic® Kit SA (Lagoa Santa, MG, Brazil) [38,39]. nHDL-C was calculated by the equation: nHDL-C=Total Cholesterol – HDL-C [40].

### Euthanasia of animals

All experimental animals were decapitated by guillotine at the end of the experiment (after seven weeks of treatment).

### Statistical analysis

Results are presented as mean ± standard deviation. One-way ANOVA was used to determine a significant difference between groups (p < 0.05) and Tukey's test to perform multiple comparisons between means.

## Results

### Content of total fructans

The total concentration of fructans found in the aqueous yacon extract was 7.96 g/100 g; 4.32 g/100 g in the unfermented product and 4.30 g/100 g in the symbiotic product. The concentrations of unfermented and synbiotic product did not show statistical difference (p < 0.05), suggesting that the microorganisms used in fermentation process were not able to modify the total fructans level.

### Body weight, feed and water intake

Table 1 shows the average consumption of water and feed as well as change in body weight throughout the experimental period; the evident increase in hunger and thirst in groups II, III and IV confirmed the induction of diabetes.

**Table 1:** Average daily intakes of water and feed and body weight of animals in non-diabetic and diabetic groups during the trial period

**Groups**	**Group I**	**Group II**	**Group III**	**Group IV**
**Parameters**				
** Water consumption (mL)**	41.26^b^ ± 0.81	173.45^a^ ± 10.63	192.16^a^ ± 9.20	183.46^a^ ± 9.16
** Feed consumption (g)**	25.47^b^ ± 0.26	38.89^a^ ± 1.20	39.56^a^ ± 1.41	38.78^a^ ± 1.31
** Body weight (g)**	306.47^b^ ± 24.33	242.41^a^ ± 8.10	245.49^a^ ± 8.23	235.29^a^ ± 6.93

Values are expressed as mean ± standard error (n=10). Means values for groups with same lowercase letter on the same line do not differ significantly at p ≤ 0.05. Group I = non-diabetic animals that received only chow diet (negative control), Group II = diabetic animals that received only chow diet (positive control), Group III = diabetic animals that received chow diet + 1 mL/kg bw/day of soybean and yacon unfermented product, Group IV = diabetic animals that received chow diet + 1 mL/kg bw/day of soybean and yacon fermented product.

It is observed that for the three variables presented in Table 1, the diabetic control group (II) did not differ statistically from the other diabetic groups (III and IV). The consumption of water and feed was greater for all diabetic rats and their weight was lower than for non-diabetic rats.

### Glycemia

In Table 2 there are the results about glucose levels.

**Table 2:** Plasma glucose levels of groups of non-diabetic and diabetic animals during the trial period

**Groups**	**Group I**	**Group II**	**Group III**	**Group IV**
**Time**				
**Glucose**				
** T0**	117.20^B.a^ ± 2.22	119.10^C.a^ ± 2.60	121.00^C.a^ ± 1.78	125.40^C.a^ ± 2.36
** T1**	129.20^AB.b^ ± 3.71	503.14^B.a^ ± 25.17	504.67^B.a^ ± 22.68	534.50^B.a^ ± 21.02
** T2**	103.36^C.b^ ± 3.97	633.00^A.a^ ± 29.43	657.57^A.a^ ± 11.31	652.67 ^A.a^ ± 9.60
** T3**	134.46^A.b^ ± 2.41	538.60^B.a^ ± 14.05	551.29^B.a^ ± 12.97	554.33^B.a^ ± 12.84

Values are expressed in mg/dL (n=10, mean ± standard error). Means values for groups with lowercase letters on the same line do not differ significantly at p ≤ 0.05; comparing times, means with same capital letters in the same column do not differ significantly p ≤ 0.05. Group I = non-diabetic animals that received only chow diet (negative control), Group II = diabetic animals that received only chow diet (positive control), Group III = diabetic animals that received chow diet + 1 mL/kg bw/day of soybean and yacon unfermented product, Group IV = diabetic animals that received chow diet + 1 mL/kg bw/day of soybean and yacon unfermented product. T0 = initial blood collection, T1 = blood collection after a week to confirm the induction of diabetes mellitus, T2 = blood collection four weeks to detect any reversal of the diabetic condition, T3 = blood collection when sacrificed at end of experiment (7 weeks).

### Liver enzymes

The AST and ALT serum levels were presented in Table 3.

**Table 3:** Concentrations of liver enzymes in non-diabetic and diabetic groups during the trial period

** Group**	**Group I**	**Group II**	**Group III**	**Group IV**
**Parameters/**				
**Time**				
** AST**				
** T0**	164.18^B.a^ ± 7.48	168.78^B.a^ ± 9.55	163.45^A.a^ ± 7.72	159.00^B.a^ ± 10.22
** T1**	156.50^B.a^ ± 9.33	159.75^B.a^ ± 15.15	180.10^A.a^ ± 11.90	175.7^AB.a^ ± 12.64
** T2**	208.09^A.a^ ± 8.24	283.90^A.a^ ± 35.68	219.36^A.a^ ± 14.85	254.20^A.a^ ± 29.05
** T3**	150.45^B.a^ ± 9.20	166.56^B.a^ ± 29.97	171.64^A.a^ ± 25.03	164.25^B.a^ ± 16.97
** ALT**				
** T0**	74.60^AB.a^ ± 3.49	78.25^B.a^ ± 2.59	73.18^B.a^ ± 2.98	67.50^C.a^ ± 1.57
** T1**	63.10^C.a^ ± 2.25	67.5^B.a^ ± 1.88	61.46^B.a^ ± 1.87	59.25^C.a^ ± 2.43
** T2**	68.90^B.b^ ± 1.75	126.80^A.a^ ± 18.05	151.63^A.a^ ± 10.80	136.80^B.a^ ± 12.38
** T3**	83.80^A.b^ ± 2.61	150.00^A.a^ ± 9.25	163.00^A.a^ ± 12.85	163.83^A.a^ ± 5.50

Values are expressed in U/L (n=10, mean ± standard error). Comparisons between groups: mean values with the same lowercase letter on the same line do not differ significantly at p ≤ 0.05, and times: equal means with capital letters in the same column do not differ significantly p ≤ 0.05. Group I = non-diabetic animals that received only chow diet (negative control), Group II = diabetic animals that received only chow diet (positive control), Group III = diabetic animals that received chow diet + 1 mL/kg bw/day of soybean and yacon unfermented product, Group IV = diabetic animals that received chow diet + 1 mL/kg bw/day of soybean and yacon unfermented product. T0 = initial blood collection, T1 = blood collection after a week to confirm the induction of diabetes mellitus, T2 = blood collection after four weeks to detected any reversal of the diabetic state, T3 = blood collection when sacrificed at end of experiment (seven weeks).

Regarding the enzyme alanine aminotransferase (ALT), there were significant differences among group I and the others, starting from time T2. Note also that all diabetic groups showed a significant increase in ALT activity after seven weeks of treatment, but noting that, once again, groups II, III and IV did not differ significantly from each other.

### Serum lipids

Table 4 shows the values of the lipid profile of the four groups throughout the experimental period.

**Table 4:** Lipid profile in non-diabetic and diabetic groups during the trial period

** Groups**	**Group I**	**Group II**	**Group III**	**Group IV**
**Parameters/**				
**Time**				
** Triglycerides**				
** T0**	135.10^B.a^ ± 12.62	173.73^B.a^ ± 11.46	156.64^B.a^ ± 12.51	175.36^C.a^ ± 13.37
** T2**	188.82^A.b^ ± 12.90	425.33^AB.a^ ± 84.89	396.18^A.a^ ± 49.70	348.50^AB.a^ ± 31.14
** T3**	149.55^AB.c^ ± 9.95	628.67^A.a^ ± 32.05	503.25^A.b^ ± 25.79	419.00^A.b^ ± 35.39
**Total cholesterol**				
** T0**	101.37^A.a^ ± 4.66	111.73 ^A.a^ ± 3.33	99.64^A.a^ ± 2.81	98.9^A.a^ ± 2.71
** T2**	80.78^B.b^ ± 3.30	100.79^A.a^ ± 4.97	102.46^A.a^ ± 3.73	100.25^A.a^ ± 4.07
** T3**	69.82^B.b^ ± 5.52	96.78^A.a^ ± 5.61	97.64^A.a^ ± 2.96	90.33^A.a^ ± 4.84
** HDL-C**				
** T0**	45.27^A.a^ ± 1.92	55.82^A.a^ ± 3.64	50.09^A.a^ ± 1.66	52.82^A.a^ ± 3.25
** T2**	43.82^A.a^ ± 2.59	52.40 ^A.a^ ± 2.20	48.64^A.a^ ± 1.50	48.70^A.a^ ± 2.25
** T3**	34.37^B.c^ ± 2.13	52.44^A.b^ ± 1.70	52.27^A.b^ ± 1.61	65.00^A.a^ ± 6.36
** nHDL-C**				
** T0**	39.56^B.a^ ± 2.35	48.57^A.a^ ± 2.81	48.63^A.a^ ± 2.15	45.50^A.a^ ± 3.01
** T2**	35.46^B.a^ ± 4.19	48.13^A.a^ ± 3.93	45.37^A.a^ ± 2.66	39.40^A.a^ ± 2.16
** T3**	56.09^A.a^ ± 3.60	55.91^A.a^ ± 3.88	49.55^A.a^ ± 2.92	46.09 ^A.a^ ± 3.19

Values are expressed in mg/dL (n=10, mean ± standard error). Means values for groups with lowercase letters on the same line do not differ significantly at p ≤ 0.05; comparing times, means with same capital letters in the same column do not differ significantly p ≤ 0.05. Group I = non-diabetic animals that received only chow diet (negative control), Group II = diabetic animals that received only chow diet (positive control), Group III = diabetic animals that received chow diet + 1 mL/kg bw/day of soybean and yacon unfermented product, Group IV = diabetic animals that received chow diet + 1 mL/kg bw/day of soybean and yacon unfermented product. T0 = initial blood collection, T2 = blood collection after four weeks to detect any reversal of the diabetic condition, T3 = blood collection when sacrificed at end of experiment (seven weeks).

It is observed that, at the end of the experiment, triglycerides levels were lower in the groups receiving treatment (III and IV) than in the positive control (group II), although these levels are higher than that observed in group I.

As for total cholesterol, it is clear that at the end of the experiment (T3), groups II, III and IV did not differ, while they were significantly different from group I.

Regarding HDL-C at the end of the experiment (T3), group IV, which consumed the potential symbiotic product, differed from all the other groups, with the highest value for this parameter. On the other hand, there was no statistical difference in non HDL-C among any of the groups during the experiment.

## Discussion

The fructans content, determined in the unfermented and symbiotic product, was found to be compatible with recommended daily intake of prebiotics (4-5 g/day) to stimulate the growth of *Bifidobacterium*[41], while doses of 3 to 10 g/day promote reduction of blood pressure, have a beneficial effect on lipid metabolism, improve gastrointestinal health [42] and provide hypoglycemic effect [43]. Although a high content of fructans in the unfermented and symbiotic products was found, a hypoglycemic effect was not observed, but it may have been one of the factors that contributed to reducing the triglycerides in groups III and IV. In addition, some studies suggest that caffeic acid, chlorogenic acid (3-caffeoylquinic acid) and very probably other caffeic acid derivatives, such as 3,4-dicaffeoylquinic, 3,5-dicaffeoylquinic and 4,5-dicaffeoylquinic acids, are also the active principles related to the hypoglycemic effect of yacon leaves [44,45].

The highest feed intake observed in the diabetic groups can be explained by the absence of circulating insulin, which causes a deficiency in glucose transport, leading to a deficiency of energy in the cells, resulting in increased feeding to compensate for that lack of energy [46].

In addition, it can be explained by physiological processes related to the pathology, such as hyperglycemia, glycosuria, and polyuria, while the weight loss is due to the catabolic processes involved in diabetes mellitus [47].

While in the non-diabetic group (group 1), the animals appeared, throughout the experiment, in good general condition, with normal appetite, progressive weight gain and maintenance of water intake, food intake and diuresis, the diabetic animals had a strong odor of urine and functional changes, such as polyuria and polydipsia. However, there were no significant changes of the coat or apathy, as described by Passos and Park [48].

In the present study, the increased serum glucose levels in groups II, III and IV confirmed that the STZ was effective in inducing diabetes mellitus, values of around 120 mg/dL rising to nearly 500 mg/dL, a level correlated with severe experimental diabetes [49].

In the blood tests used to check for induction reversal (T2), the highest blood glucose levels were recorded, thus confirming the absence of reversion. The rate of induction was 100%, since long-term diabetes mellitus was found in all animals, as observed by Rakieten et al. [50], in rats and dogs, at the same doses of 50 mg STZ per kg body weight.

At T0, there was no statistical difference in glycemic level among the groups. After induction and until the end of the experiment, only group I (non-diabetic) maintained low values, by significantly differing from the other groups, which all had become diabetic.

Although a reduction in blood glucose occurred at the end of the experiment, it cannot be suggested that, thus revealed a tendency for diabetes to stabilize, owing to the presence of yacon and/or probiotic bacteria, since the animals in group II received only water and feed and showed the same reduction. Studies by Maffezzoli [51] showed results similar to those in this study.

The organism itself tends to normalize high blood glucose levels by three main routes: stimulating glucose uptake by peripheral tissues (muscle and adipose tissue); altering the insulin metabolism (by reducing the degradation of insulin in the liver or stimulating insulin secretion) and, finally, by inhibiting the reabsorption of glucose by the kidneys, resulting in the elimination of glucose in the urine [52].

The glycemic control observed in animal studies and early studies in humans has indicated that ingested fructooligosaccharides probably act to stimulate glucose utilization in peripheral tissues (muscle and adipose tissue) [52].

The enzymes analyzed in this study are markers of liver injury; the observed increase in AST and ALT activity indicates an aggressive hepatocyte injury [53] with regard to these enzymes, it was noted that there was no significant difference among the three diabetic groups, indicating again that the presence of yacon extract did not improve the condition of the liver of animals in groups III and IV.

A study by Baroni et al. [54] evaluated the activity of AST and ALT in the plasma of diabetic and non-diabetic animals that received 10% hydroethanolic extract of yacon for 14 days. These researchers observed that the activity of these enzymes increased in diabetic rats but that, on administration of the extract, the enzyme activities of diabetic rats were close to those of control animals. One possible explanation is that the administration of the yacon extract may have decreased the hepatic lesions caused by the disease. However, these results were not confirmed in our study.

Regarding the lipid profile, it was observed that at the end of the experiment, triglycerides levels were lower in groups III and IV than in group II, although all these levels were higher than that observed in group I, indicating that both the unfermented and fermented products may have reduced this serum lipid in STZ-induced animals. Numerically, group IV had the lowest triglycerides, which may be related to the aqueous yacon extract [55] and the probiotic one [56] used in the synbiotic product.

Whereas diabetes results in increased lipolysis in adipose tissue, leading to higher blood levels of fatty acids, there is also a greater production of ketone bodies by the liver. However, the excess fatty acid captured by the liver is not fully oxidized to ketone bodies by ketogenesis. Thus, these excess fatty acids are directed to the synthesis of triglycerides, which is converted into VLDL. As VLDLs in excess are not be fully metabolized by lipoprotein lipase, a state of a hypertriglyceridemia would occur [57].

As for total cholesterol, note that at the end of the experiment groups II, III and IV did not differ, although they were significantly different from group I. However, group IV again showed, numerically, the lowest value for this parameter, although not statistically different from the others; that may be an indicative of a tendency, in agreement with the results observed by Rossi et al. [58], which showed that *E. faecium* CRL 183 was able to reduce total cholesterol levels by 18.4% in hyperlipidemic rabbits.

A variety of past *in vitro* experiments and *in vivo* trials have provided evidence to support the roles of probiotics in lowering serum cholesterol and improving lipid profiles.

Several mechanisms of cholesterol reduction by probiotics via control of cholesterol metabolism have been proposed. One such mechanism is the removal of cholesterol by assimilation. The assimilation of cholesterol by probiotics in the small intestine could reduce serum cholesterol by reducing the absorption of cholesterol in the intestines [59]. Probiotics must be viable and growing, in order to be able to remove or assimilate cholesterol [60]. Other researchers have suggested that the incorporation of cholesterol into cell membranes could be another mechanism used to reduce cholesterol in media, or that it involves the ability of certain probiotics to deconjugate bile acids enzymatically [61].

Regarding HDL-C at the end of the experiment (T3), group IV (symbiotic) differed from the other groups, with the highest value for this parameter, which may be due to the presence of *E. faecium* CRL 183. This ability of *E. faecium* CRL 183 to promotion increase in HDL-C has been observed in a study by Rossi et al. [58], where the authors showed an increase of this fraction by 17.8%.

In comparison to the results of Khamisy [62], who administered a suspension of *Bifidobacterium* and *Lactobacillus acidophilus*, separately or in combination to diabetic albino rats, the presented results were more satisfactory in terms of the increase in HDL-C in group IV, which received the symbiotic product; this is an important condition in reducing the risk of onset of coronary heart disease in diabetics.

In another study conducted by Manzoni et al. [17], concerning the beneficial effects on rat serum lipid effects of soy yogurt fermented with *E. faecium* CRL 183, only HDL levels were changed positively, by showing a 46% increase.

Research indicates that the hypocholesterolemic action of probiotics may be enhanced by the use of a prebiotic [63]. The effects of probiotic combinations and certain prebiotics i.e. symbiotic on blood lipids were investigated by Kiebling et al. [64] and Greany et al. [65]. One study reported that the ingestion of yogurt fermented with *L. acidophilus* 145 and *B. longum* 913 plus 1% oligofructose [64] raised the level of HDL-C, while another, which tested the combination *L. acidophilus* DDS-1, *B. longum* UABL-14 and fructooligosaccharides, found no effect [65].

Regarding the fraction nHDL-C, no statistical difference was observed among the 4 groups during the experimental period.

## Conclusion

The product based on aqueous extracts of yacon and soybean fermented or not with *Enterococcus faecium* CRL 183 and *Lactobacillus helveticus* ssp *jugurti* 416, showed no positive effect on blood glucose levels, but was effective in reducing triglycerides both unfermented and symbiotic products and increasing HDL-cholesterol (symbiotic product alone).

## Abbreviations

AST: Aspartate aminotransferase; ALT: Alanine aminotrasferase; HDL-C: High density lipoprotein cholesterol; n-DHL-C: Non high density lipoprotein cholesterol; VLDL: Very low density lipoprotein; STZ: Streptozotocin.

## Competing interests

The authors declare that they have no competing interests.

## Authors’ contributions

MNR: was involved in design, data collection, drafting the manuscript and revising it critically for important intellectual content. NDPS, DCUC, LSC, RCV, RAP: participated in data collection, interpretation of results and drafting the manuscript. EAR: was involved in design, drafting the manuscript and revising it critically for important intellectual content. All authors read and approved the final manuscript.

## References

[B1] Bransome ED Financing the care of diabetes mellitus in the U.S Diabetes Care 1992 15 1 5 10.2337/diacare.15.1.1 1559412

[B2] Negri G Diabetes melito: plantas e princípios ativos naturais hipoglicemiantes Revista Brasileira de Ciências Farmacêuticas 2005 41 2 121 142

[B3] Robbins SL Cotran RS Kumar V Taranto G Patologia estrutural e funcional 1991 4 Rio de Janeiro: Guanabara Kogan 817 826

[B4] Shoelson SE Insulin and other antidiabetic agents Kirk-Othmer encyclopedia of chemical technology 1995 3 New York: John Wiley 662 676

[B5] Berne RM Genuth SM Fisiologia 2000 4 Rio de Janeiro: Guanabara Koogan 190

[B6] Godoy P Bogliolo L Pâncreas Endócrino Patologia 2000 6 Rio de Janeiro: Guanabara Koogan 1004 1008

[B7] Said O Khalil K Fulder S Azaizeh H Ethnopharmacological survey of medicinal herbs in Israel, the Golan heights and the West Bank Region J Ethnopharmacol 2002 3 251 265 12426094 10.1016/s0378-8741(02)00253-2

[B8] Mori DM Baviera AM De Oliveira RLT Vendramini RC Brunetti IL Pepato MT Temporal response pattern of biochemical analytes in experimental diabetes Biotechnol Appl Biochem 2003 38 183 191 10.1042/BA20030034 12826018

[B9] Ludke MC López J Colesterol e composição dos ácidos graxos nas dietas para humanos e na carcaça suínas Ciência Rural 1999 29 1 181 187 10.1590/S0103-84781999000100033

[B10] Armaganijan D Batlouni M Impacto f traditional risk factors Revista da sociedade de cardiologia do Estado de São Paulo 2000 10 São Paulo: Edson Stefanini, SOCESP 689 693

[B11] Rifai N Warnick GR Burtis CA, Ashwood ER, Bruns DE Lipids, lipoproteins, apolipoproteins, and other cardiovascular risk factors Tietz textbook of clinical chemistry and molecular diagnostics 2006 40 St. Louis: Elsevier Saunders 903 981

[B12] FAO/WHO Evaluation of health and nutritional properties in food including powder milk with live lactic acid bacteria Expert consultation report 2011 Cordoba http://www.fao.org/es/ESN/Probio/probio.htm

[B13] Rossi ER Desenvolvimento e avaliação biológica do potencial hipocolesterolêmico de um novo produto probiótico de soja Tese (livre docência)-faculdade de ciências farmacêuticas 2000 Araraquara: Universidade Estadual Paulista

[B14] Rossi EA Vendramini RC Carlos IZ Ueiji IS Squinzari MM Silva Junior SI Valdez GF Effects of a novel soy product on the serum lipids of hypercholesterolemic rabbits Arq Bras Cardiol 2000 74 213 216 10.1590/S0066-782X2000000300003 10951824

[B15] Rossi EA Vendramini RC Carlos IZ Oliveira MG Valdez GF Efeito de um novo produto fermentado de soja sobre os lípides séricos de homens adultos normocolesterolêmicos Arq Latin Nutr 2003 53 47 51 12942871

[B16] Rossi EA Rosier I Dâmaso AR Carlos IZ Vendramini RC Abdalla DSP Talarico VH Minto DF Determinação de isoflavonas nas diversas etapas do processamento de “iogurte” de soja Alim Nutr 2004 15 2 93 99

[B17] Manzoni MSJ Rossi EA Carlos IZ Vendramini RC Duarte ACGO Tenorio NM Amorim DB Damaso AR Fermented soy product supplemented with isoflavones affects adipose tissue in a regional-speccific manner and improves HDLcholesterol in rats fed on a cholesterol-enriched diet Eur Food Res Technol 2008 227 6 1591 1597 10.1007/s00217-008-0883-1

[B18] Rivera D Manrique Zumo de yacón - ficha técnica Centro internacional de la Papa (CIP) 2005 Lima: Disponível em http://www.cipotato.org/artc/cipcrops/fichazumoyacon.pdf

[B19] Aybar MJ Sánchez Riera AN Grau A Sánchez SS Hipoglycemic effect of the water extract of smallantus sonchifolius (yacon) leaves in normal and diabetic rats J Ethnopharmacol 2001 74 2 125 132 10.1016/S0378-8741(00)00351-2 11167030

[B20] Pereira D Gibson GR Effects of consumption of probiotics and prebiotics on serum lipid levels in humans Critical Rev Biochem Molec Biol 2002 37 4 259 281 10.1080/10409230290771519 12236466

[B21] Matsuzaki T Yamazaki R Hashimoto S Yokokura T Antidiabetic effects of an oral administration of lactobacillus casei in a non-insulin-dependent diabetes mellitus (NIDDM) model using KK-Ay mice Endocr J 1997 44 357 365 10.1507/endocrj.44.357 9279510

[B22] Matsuzaki T Nagata Y Kado S Uchida K Hashimoto S Yokokura T Effect of oral administration of lactobacillus casei on alloxan-induced diabetes in mice APMIS 1997 105 637 642 10.1111/j.1699-0463.1997.tb05065.x 9298103

[B23] Tabuchi M Ozaki M Tamura A Yamada N Ishida T Hosoda M Hosono A Antidiabetic effect of lactobacillus GG in streptozotocin-induced diabetic rats Biosci Biotechnol Biochem 2003 67 1421 1424 10.1271/bbb.67.1421 12843677

[B24] Cani PD Neyrinck AM Fava F Knauf C Burcelin RG Tuohy KM Gibson GR Delzenne NM Selective increases of bifidobacteria in gut microflora improve high-fat-diet-induced diabetes in mice through a mechanism associated with endotoxaemia Diabetologia 2007 50 2374 2383 10.1007/s00125-007-0791-0 17823788

[B25] Yadav H Jain S Sinha PR Antidiabetic effect of probiotic dahi containing lactobacillus acidophilus ans lactobacillus casei in high fructose fed rats Nutr 2007 23 62 68 10.1016/j.nut.2006.09.002 17084593

[B26] Yun SI Park HO Kang JH Effect of lactobacillus gasseri BNR17 on blood glucose levels and body weight in a mouse model of type 2 diabetes J Appl Microbiol 2009 107 5 1681 1686 10.1111/j.1365-2672.2009.04350.x 19457033

[B27] Lye HS Kuan CY Ewe JA Fung WY Liong MT The improvement of hypertension by probiotics: effects on cholesterol, diabetes, renin and phytoestrogens Int J Mol Sci 2009 10 3755 3775 10.3390/ijms10093755 19865517 PMC2769158

[B28] Vendramini AP Efeito da ingestão de um produto de soja fermentado com enterococcus faecium e lactobacillus helveticus Dissertação (mestrado em análises clínicas)-faculdade de ciências farmacêuticas 2002 Araraquara: Universidade Estadual Paulista

[B29] Bedani R Rossi EA Lepera JS Wang CC Valdez GF Efeito de um novo produto fermentado de soja, enriquecido com isoflavonas e cálcio, sobre o tecido ósseo de ratas Arc Latinoam Nutr 2006 56 146 152 17024959

[B30] Shiguemoto GE Rossi EA Baldissera V Gouveia CH Valdez GMF Perez SEA Isoflavone-supplemented soy yoghurt associated with resistive physical exercise increase bone mineral density of ovariectomized rats Maturitas 2007 57 261 270 10.1016/j.maturitas.2007.01.011 17368767

[B31] Rossi EA Cavallini DCU Carlos IZ Vendramini RC Dâmaso AR Valdez GF Intake of isoflavone-supplemented soy yogurt fermented with enterococcus faecium lowers serum total cholesterol and non-HDL cholesterol of hypercholesterolemic rats Eur Food Res Technol 2008 228 2 275 282 10.1007/s00217-008-0932-9

[B32] Cavallini DCU Efeito do “iogurte” de soja fermentado com E. faecium e suplementado com isoflavonas nos marcadores laboratoriais de risco cardiovascular e na aterosclerose140f. relatório (pós-doutorado) – faculdade de ciências farmacêuticas 2009 Araraquara: UNESP

[B33] Pauly-Silveira ND O emprego da metodologia de resposta no desenvolvimento de um novo produto simbiótico, fermentado com enterococcus faecium CRL 183 e lactobacillus helveticus ssp jugurti 416, à base de extratos aquosos de soja e de yacon (smallanthus sonchifolius) UNESP, dissertação (mestrado em alimentos e nutrição - área de ciência dos alimentos) - faculdade de ciências farmacêuticas 2009 Araraquara - SP: Universidade Estadual Paulista

[B34] Yoruk M Kanter M Meral I Agaoglu Z Localization of glycogen in the placenta and fetal and maternal livers of cadmium exposed diabetic pregnant rats Biol Trace Elem Res 2004 96 217 226 14716101 10.1385/BTER:96:1-3:217

[B35] Kanter M Yoruk M Koc A Meral I Karaca T Effects of cadmium exposure on morphological aspects of pancreas, weights of fetus and placenta in streptozotocin-induced diabetic pregnant rats Biol Trace Elem Res 2003 93 189 200 10.1385/BTER:93:1-3:189 12835501

[B36] Muir JG Shepherd SJ Rosella O Rose R Barrett JS Gibson PR Fructan and free fructose content of common Australian vegetables and fruit J Agric Food Chem 2007 55 16 6619 6627 10.1021/jf070623x 17625872

[B37] Lerco MM Spadella CT Machado JLM Schellini SA Padovani CR Experimental alloxan diabetes induced: a model for clinical and laboratory studies in rats Acta Cir Bras 2003 18 2 132 142 [online]

[B38] Trinder R Determination of glucose in blood using glucose with alternative oxygen acceptor Ann Clin Biochem 1969 6 27 27

[B39] Sacks DB Bruns DE Goldstein DE Maclaren NK Mcdonald JM Parrot M Guidelines and recommendations for laboratory analysis in the diagnosis and management of diabetes mellitus Clin Chem 2002 48 436 472 11861436

[B40] Lu W Resnick HE Jablonski KA Jones KL Jain AK Howard WJ Robbins DC Howard BV Non-HDL cholesterol as a predictor of cardiovascular disease in type 2 diabetes: the strong heart study Diabetes Care 2003 26 1 16 23 10.2337/diacare.26.1.16 12502653

[B41] Desjardins ML Roy D Goulet J Growth of bifidobacteria and their enzyme profiles J Dairy Sci 1990 73 299 307 10.3168/jds.S0022-0302(90)78673-0

[B42] ADA (American Dietetic Association) Position of the American dietetic association: functional foods J Am Diet Assoc 1999 99 1278 1285 10524397 10.1016/S0002-8223(99)00314-4

[B43] Quinteros ETT Produção com tratamento enzimático e avaliação do suco de yacon Tese (doutorado em tecnologia de alimentos) – faculdade de engenharia de alimentos 2000 Campinas: Universidade Estadual de Campinas

[B44] Genta SB Cabrera WM Mercado MI Grau A Catalán CA Sánchez SS Hypoglycemic activity of leaf organic extracts from smallanthus sonchifolius: constituents of the most active fractions Chem Biol Interact 2010 185 2 143 152 10.1016/j.cbi.2010.03.004 20211156

[B45] Xiang Z He F Kang TG Dou DQ Gai K Shi YY Kim YH Dong F Anti-diabetes constituents in leaves of smallanthus sonchifolius Nat Prod Commun 2010 5 1 95 98 20184030

[B46] Wong KK Tzeng ES Appearance of different diabetic symptoms after streptozocin administration: a comparison study Biochem Mol Biol Int 1993 30 1035 1041 8220250

[B47] Meigs JB Hu FB Rifai N Manson JE Biomarkers of endothelial dysfunction and risk of type 2 diabetes mellitus J Am Med Assoc 2004 291 16 1978 1986 10.1001/jama.291.16.1978 15113816

[B48] Passos LML Park YK Frutooligossacarídeos: implicações na saúde humana e utilização em alimentos Ciência Rural 2003 33 2 385 390 10.1590/S0103-84782003000200034

[B49] Grover JK Vats V Rathi SS Anti-hyperglycemic effect of eugenia jambolana and tinospora cordifolia in experimental diabetes and their effects on key metabolic enzymes involved in carbohydrate metabolism J Ethnopharmacol 2000 73 3 461 470 10.1016/S0378-8741(00)00319-6 11091000

[B50] Rakieten N Rakieten L Nadkarni M Studies on the diabetogênico action of streptozotocin Cancer Chemother Rep 1963 29 91 98 13990586

[B51] Maffezzoli AS O efeito da insulina sobre concentrações glicêmicas e lipídicas em ratos normais e diabéticos induzidos pela estreptozotocina Trabalho de conclusão de curso 2004 Santa Catarina: Universidade do Vale do Itajaí, UNIVALI

[B52] Silva ADS A raiz yacon (smallanthus sonchifolius poepping & endlicher) como fonte de fibras alimentares, sua caracterização físico-quimica, uso na panificação e sua influência na glicemia pós–prandial Tese (doutorado) - departamento de ciência e tecnologia de alimentos 2007 Florianópolis: Universidade Federal de Santa Catarina

[B53] Ali MM Agha FG Amelioration of streptozotocin-induced diabetes mellitus, oxidative stress and dyslipidemia in rats by tomato extract lycopene Scand J Clin Lab Invest 2009 69 3 371 379 10.1080/00365510802658473 19148834

[B54] Baroni S Kemmelmeier FS Caparroz-Assef SM Cuman RKN Bersani-Amado CA Effect of crude extracts of leaves of smallanthus sonchifolius (yacon) on glycemia in diabetic rats Braz J Pharma Sci 2008 44 3 521 530

[B55] Kaur IP Chopra K Saini A Probiotics: potential pharmaceutical applications Eur J Pharma Sci 2002 15 1 9 10.1016/S0928-0987(01)00209-3 11803126

[B56] Koop-Hoolihan L Prophylactic and therapeutic uses of probiotics: a review J Am Diet Assoc 2001 101 229 238 10.1016/S0002-8223(01)00060-8 11271697

[B57] Contreras F Rivera M Vásquez JF YáneZ CJB De La Parte MA Velasco M Diabetes e hipertensión aspectos clínicos y terapêuticos Arch Vzlanos Farmacol Terap 2000 19 9 24

[B58] Rossi EA Carlos IZ Vendramini RC Machado CO Cyrillo RNS Perazzo FF Valdez GF Avaliação do potencial alergênico de um novo produto fermentado de soja RBCF Ver Brás Ciênc Farm São Paulo 2000 21 1 103 113

[B59] Pigeon RM Cuesta EP Gilliland SE Binding of free bile acids by cells of yogurt starter culture bacteria J Dairy Sci 2002 85 2705 2710 10.3168/jds.S0022-0302(02)74357-9 12487437

[B60] Liong MT Shah NP Acid and bile tolerance and the cholesterol removal ability of bifidobacteria strains Biosci Micro 2005 24 1 10 10.3168/jds.S0022-0302(05)72662-X 15591367

[B61] Liong MT Shah NP Roles of probiotics and prebiotics on cholesterol: the hypothesized mechanisms Nutrafoods 2005 4 45 57

[B62] Khamisy AESE Effect of bifidobacterium and lactobacillus acidophilus in diabetic rats The 5th Arab and 2nd international: annual scientific conference 2010 Egypt: Faculty Specific Education - Mansoura University 2425 2439

[B63] Zhang F Xiaomim H Xiaobing F Guije L Hong Y Selection and optimization procedure of symbiotic for cholesterol removal Anaerobe 2007 13 185 192 10.1016/j.anaerobe.2007.06.001 17681806

[B64] Kiebling G Schneider J Jahreis G Longterm consuption of fermented dairy products over 6 months increases HDL cholesterol Eur J Clin Nutr 2002 56 843 849 10.1038/sj.ejcn.1601399 12209372

[B65] Greany KA Bonorden MJ Hamilton-Reeves JM McMullen MH Wangen KE Phipps WR Feirtag J Thomas W Kurzer MS Probiotic capsules do not lower plasma lipids in young women and men Eur J Clin Nutr 2008 62 232 237 10.1038/sj.ejcn.1602719 17356554

